# COVID-Related Concerns, the Need for Help, and Perceived Microaggression among Young Ultra-Orthodox Jewish Respondents in Israel

**DOI:** 10.3390/ijerph18126445

**Published:** 2021-06-14

**Authors:** Faiga Weiden, Michal Levinsky, Miriam Schiff, Nati Becker, Ruth Pat-Horenczyk, Rami Benbenishty

**Affiliations:** 1 Ministry of Welfare and Social Services, 2 Kaplan Street, Jerusalem 9100801, Israel; FaigaW@molsa.gov.il; 2 School of Social Work and Social Welfare, Hebrew University of Jerusalem, Jerusalem 9190501, Israel; michal.levinsky@mail.huji.ac.il (M.L.); nati.becker@mail.huji.ac.il (N.B.); ruth.pat-horenczyk@mail.huji.ac.il (R.P.-H.); ramibenben@gmail.com (R.B.); 3 Department of Education and Social Sciences, Andrés Bello National University, Santiago 7591538, Chile

**Keywords:** COVID-19, ultra-Orthodox Jews, microaggression, mitigation guidelines, COVID-related concerns

## Abstract

Minority groups are especially vulnerable to the negative psychological and economic consequences of the COVID-19 pandemic. This study focused on one prominent minority group in Israel: ultra-Orthodox Jews. It examined the rate of exposure to COVID-19, adherence to COVID-19 mitigation guidelines, difficulties with adherence to COVID-19 guidelines, COVID-related concerns, financial hardships, the need for help, and microaggression during the first wave of the pandemic (April–May 2020). It then examined multivariate prediction of COVID-related concerns, the need for help, and microaggression. The sample comprised 252 respondents, with 67% female and a mean age of 32.85 (SD = 10.63). Results showed that 78.8% of the participants knew at least one person who had tested positive for COVID-19, and 31.4% knew at least one person who had passed away from COVID-19. Only 59.7% of the participants reported high adherence to social distancing guidelines. Perceived microaggression was predicted by the difficulties with adherence to COVID-19 guidelines, the level of stress associated with exposure to the media, and financial hardships. The study’s implications point to the centrality of perceived microaggression and the necessity of adopting culturally sensitive approaches to engage minorities in public efforts to fight the spread of viruses.

## 1. Introduction

By the end of March 2021, the COVID-19 pandemic had infected nearly 130 million people and caused the death of more than 2.8 million worldwide [1]. This study was conducted during the first wave of the COVID-19 pandemic in Israel, April–May 2020, when the number of positive cases was between 15,500 and 17,000, with the number of deaths reaching close to 300 by the end of May [2].

Although the entire global population is susceptible to COVID-19, minority groups are especially vulnerable, both to becoming infected by the virus and to experiencing its negative psychological and economic consequences [3,4]. In Israel, the two major cultural minorities are Arabs and ultra-Orthodox Jews. This study addressed the ultra-Orthodox Jews.

### 1.1. A Brief Description of the Ultra-Orthodox Jews in Israel

Ultra-Orthodox Jews in Israel comprise 13% of the Israeli population [5]. They are defined as a community that is dedicated to strict observance of a series of Jewish laws known as MITZVOT (Hebrew). These laws cover all life domains, including gender segregation, distinct outfits, sexual relations, and purity rituals. Ultra-orthodox Jews also hold distinct gender roles; men are expected to study Jewish laws and commentaries throughout their lives as their major occupation, while women are expected to be responsible for household chores, raising children, and taking care of the family budget [6]. They are also characterized by low socio-economic status, higher dependency on public transportation, and large families [7]. The average number of children per family is 7.1, compared with 2.7 children in the general population [8]. Most of these large families live in small apartments in buildings with many similar families. They live in separate towns or neighborhoods, mainly to preserve their unique identity in light of the perceived constant threat from secular society [5]. This group has an active community life with most of their daily social interactions and social life occurring within their crowded living environments [9]. Living with large families in small apartments and interacting constantly with members of the same community raises the probability of large-scale COVD-19 contagion.

The present study addressed a list of risk factors for psychological distress among ultra-Orthodox Jews during the COVID-19 pandemic: level of exposure to COVID-19, compliance with governmental mitigation guidelines and difficulties in compliance with those guidelines, exposure to media coverage and the stress associated with such exposure, and financial hardships during the pandemic. Given the distinctive aspects of the COVID-19 crisis and the call for conceptualizing the crisis within a trauma perspective and measuring its consequences differently than previous collective traumatic events [10], we assessed psychological distress by COVID-related concerns, the need for help, and perceived microaggression, which will be further explained below.

### 1.2. Level of Exposure to COVID-19

COVID-19 has hit the ultra-Orthodox Jewish community hard around the globe. In New York, for example, COVID-19 caused the death of influential religious leaders and tore apart families at a rate that exceeded other ethnic or religious groups [11]. In Israel, ultra-Orthodox Jews were acknowledged as a vulnerable group at risk for contracting COVID-19 [12]. Although they comprise 13% of the population, ultra-Orthodox Jews accounted for more than one-third of confirmed cases of COVID-19 and 60–70% of Israel’s COVID-19 hospitalizations [13]. Furthermore, ultra-Orthodox towns constitute 6% of all the towns in Israel, while the rate of COVID-19 cases in these towns was 20.5% and kept rising [14]. Although the death toll among this population was low at the beginning of the pandemic, it later became twice as high as in the general Jewish population [5]. Nevertheless, many ultra-Orthodox were convinced that they could continue with religious activities that were important to them, such as praying in public and sending children to crowded schools, thereby challenging the social distancing mitigation guidelines [15]. Such continuously high exposure was a risk factor for psychological distress [16,17]. This study addressed the level of exposure to COVID-19 as one of the risk factors for psychological distress.

### 1.3. Adherence to Governmental Mitigation Guidelines and Difficulties in Compliance

The Israeli government’s emergency mitigation guidelines collided with fundamental aspects of ultra-Orthodox Jewish life, such as praying indoors in a quorum (*minyan*) of ten men, or assembling groups for Torah study, requisite activities that exceeded the number of persons allowed by health authorities to congregate indoors. Community rabbinical authorities demanded that such traditions be maintained at any cost [5], creating conflicting feelings among many members of the ultra-Orthodox community about whether to adhere to the government mitigation guidelines or obey their revered rabbis. This community tends to distrust governmental authorities to begin with (Slobodin and Cohen, 2020), and tends to react favorably to no coercive changes and guidelines that are reached through negotiation from their reliable sources (Tikotsky et al., 2020). However, no negotiation between the government and representatives of this community took place during the period of this study.

Adherence to governmental social distancing guidelines was also difficult to maintain due to living conditions. For example, maintaining quarantine in large, multigenerational households in small apartments is highly challenging [18]. We could not find any study examining those challenges. In this study, we examined the level of adherence to the governmental mitigation guidelines, the extent of difficulty each of these guidelines created, and the associations of these difficulties with psychological distress during the first months of the pandemic.

### 1.4. Exposure to Media Coverage of COVID-19 and the Stress Related to Media Coverage

Due to religious and community norms, many ultra-Orthodox Jews are considerably less exposed to digital technology like the Internet, smartphones, and television than the general Israeli population. Religious leaders in this society tend to restrict the use of the Internet and other social media forms because they perceive them as a threat to the society’s values [19,20]. Nevertheless, more than 60% of the adult ultra-Orthodox population does use the Internet [8], although mainly for work-related matters [20]. This study relates to the portion of the ultra-Orthodox population with access to the Internet. Therefore, it is aware of its inability to generalize its findings to Israel’s entire ultra-Orthodox Jewish community. Preliminary evidence from studies conducted during the pandemic suggests that exposure to media coverage of COVID-19 is associated with increased psychological distress [21,22], even after adjusting for background variables and actual exposure to COVID-19 [22]. It is not clear, however, to what extent these findings should be generalized to ultra-Orthodox Jews, as the exposure to media studies were conducted on the general population in other countries [23]. A previous study conducted by the authors among a large sample of Israeli higher education students revealed that stress related to media coverage of COVID-19 was a greater risk factor for psychological distress than the actual level of exposure to media coverage of the pandemic (Authors, in press). This finding may be particularly relevant to ultra-Orthodox society, as the media kept covering their continuous violation of COVID-19 mitigation guidelines (e.g., [24]). Thus, the current study explored both levels of exposure to media coverage of COVID-19 and media-related stress as two potential risk factors for psychological distress among ultra-Orthodox Jews.

### 1.5. Financial Hardships Due to COVID-19 

The COVID-19 pandemic is known not only as a health crisis but also as a global financial crisis [25]. COVID-19 in Israel hit the ultra-Orthodox Jewish population, especially males, more than the general male Jewish population. Between March to May 2020—the approximate time the study was conducted—the employment rate among ultra-Orthodox Jews dropped by 35% (34% among males and 37% among females) compared with a similar time during 2019, whereas the decline in the employment rate among the general Jewish population was, at that period, around 20% (19% among males and 27% among females) (The Israel Democracy Institute press release, 2020). Moreover, given that this population is characterized by low rates of formal labor force participation, many were not entitled to the stimulus grants and other types of economic assistance provided by the Israeli government [7].

Associations between economic hardship during COVID-19 and greater psychological distress have been found in other contexts and populations [26]. Nonetheless, it is expected that economic hardships, and especially economic deterioration during the pandemic, would serve as another risk factor for psychological distress among the study population.

## 2. Psychological Distress during COVID-19: Concerns, the Need for Help, and Perceived Microaggression

Many of the studies conducted on the psychological consequences of COVID-19 have addressed mental health indicators, such as anxiety and depression. This study focused on other aspects of distress: COVID-related concerns, a perceived need for professional help, and reported level of microaggression.

*COVID-related concerns*. The distinctive aspects of the COVID-19 pandemic call for forming innovative approaches for conceptualizing distress [10]. Therefore, rather than focusing on mental health distress and symptoms of depression [27] and anxiety [28], the present study constructed a new measure of concern that was tailored specifically for the ultra-Orthodox population. This included concern for personal and family health, which had the potential to be high due to the high spread of COVID-19 among this group. It also included concerns about their distancing from extended family and from their community, crucial aspects of the ultra-Orthodox way of life, which could have served as a great source for concern [29].

*Perceived need for professional help*. A person’s conscious decision that he or she needs professional help is considered a part of help-seeking behavior [30]. A perceived need for help was associated with a higher level of psychological distress in other populations [31,32]. Examining the rate of perceived need for professional help and its associations with psychological distress is especially important because the prevalence of certain types of mental health difficulties, such as OCD and eating disorders, among the ultra-Orthodox population is higher than among the general population [33]. The present study further explored the rate of perceived need for professional help and factors associated with that need in a population that, to our best knowledge, has not been addressed before in this regard; namely, Israeli ultra-Orthodox Jews. 

*Microaggression*. This term is defined as brief, subtle acts, whether verbal, behavioral, or environmental, intentional or unintentional, aimed at racial or ethnic minorities signaling degrading and humiliating messages to individuals about their membership in a particular racial or ethnic group [34]. Microaggression is associated with mental health distress, including a higher level of depressive symptoms and lower well-being [35,36].

Microaggression was associated with negative affect among all minority groups [37]. In Israeli society, microaggression is said to make minority groups feel transparent and powerless [38]. During COVID-19, the ultra-Orthodox population in Israel experienced many forms of microaggression in the media, from governmental officials, and in informal encounters [39]. The background for the growing expression of microaggression was the relatively low adherence by ultra-Orthodox Jews to COVID-19 mitigation guidelines at the beginning of the pandemic. Their behavior was interpreted as highly disrespectful to the general society and endangering the health of the general public. It triggered accusations against the ultra-Orthodox population as a whole as being transmitters of the virus [8]. People from “red” (i.e., an expression used by the Ministry of Health to designate neighborhoods or towns with high levels of COVID-19 infection) ultra-Orthodox towns such as Bnei Brak were allegedly treated differently upon entry to health services, e.g., when women were admitted to a hospital maternity ward [40]. The present study examined microaggression rates toward the ultra-Orthodox population during the COVID-19 pandemic as perceived by that community, as well as the risk factors for microaggression.

### Research Questions

1. What are the rates of exposure to COVID-19, levels of adherence to COVID-19 mitigation guidelines, difficulties with adherence to these guidelines, COVID-related concerns, economic hardships, the need for help, and experience of microagression among ultra-Orthodox Jews?
2. What are the associations between each of the following risk factors: level of exposure to COVID-19, media coverage, media coverage-related stress, adherence to COVID-19 mitigation guidelines, and difficulties with guidelines, on the one hand, and on the other hand, COVID-related concerns, feeling the need for help, and reported microaggression among ultra-Orthodox Jews?
3. To what extent are COVID-related concerns, the need for help, and reported microaggression among ultra-Orthodox Jews predicted by the level of exposure to COVID-19 infection, media coverage, media coverage-related stress, adherence to COVID-19 mitigation guidelines, and difficulties with guidelines, after controlling for background variables?

## 3. Methods

### 3.1. Research Population

The research population was comprised of young ultra-Orthodox Jewish families with access to email or smartphones. Around 400,000–450,000 people are defined as ultra-Orthodox in Israel at the relevant ages [8]. Among them, approximately 60% have access to the Internet or smartphones [8]. The current study, therefore, relates to a population of about 260,000 people. They live in ultra-Orthodox Jewish communities and thus can be described as mainstream ultra-Orthodox. Many of the men were *avrechim*, meaning they allocated most of their days to learning the Torah (religious teachings) and perceived themselves to be part of *frum* (religiously observant) society. Their children attended schools belonging to the ultra-Orthodox Jewish educational stream 

### 3.2. Sample

In this study, we recruited the respondents using a convenience sampling method. We approached 3550 people via the mailing list of an Ultra-Orthodox community center and ten different network groups of Ultra-Orthodox Jews. A total of 346 people from these sources responded to an online questionnaire (response rate of 9.7%); however, 86 did not respond to any of the background variables, two did not provide information regarding their gender, and six respondents did not define their religiosity level (i.e., whether they were ultra-Orthodox Jews or not). These 94 were removed from the analyses. Overall, then, the sample comprised 252 respondents: 67% female, with a mean age of 32.85 (SD = 10.63). Considering the research population comprises about 260,000 people, the percentage of the sample size from the total population is approximately 0.097%. The participants’ average number of children was 3.41 (SD = 2.91) with a range of 0–12. As for formal educational level, 8.2% had completed elementary or high school, 21.0% had completed *yeshiva* (a higher education religious institute), 22.6% had completed a teaching seminary (a post-high school educational institute for ultra-Orthodox girls), and 48.2% held a higher education degree (at least a bachelor’s degree). Respondents were recruited through a combination of convenience and snowball sampling methods.

### 3.3. Data Collection and Ethical Considerations

Following approval by the university ethics committee, the online questionnaire was distributed via a link to private emails or WhatsApp groups known to two of the authors by family ties, friendships, or employment places. Each respondent was requested to distribute an online link to friends, family members, or colleagues at work who defined themselves as ultra-Orthodox. The questionnaire was answered anonymously. Informed consent was asked for on the first-page screen, and only participants who marked “agree” were referred to the questionnaire. Data were collected between 30 April and 19 May 2020, from the middle to almost the end of the pandemic’s first wave in Israel. During this period, there were several lockdowns throughout the country during Jewish holidays. Additionally, several ultra-Orthodox towns and neighborhoods experienced long periods of lockdown. 

### 3.4. Measurements

The measurement tools were developed specifically for this study to ensure they were appropriate for the unique situation of the global pandemic and culturally sensitive to the ultra-Orthodox population in Israel.

### 3.5. Independent Variables

***Exposure to COVID-19*** was measured by four questions: (a) “Since the beginning of the COVID-19 pandemic, were you quarantined due to infection or suspected infection?”; (b) “Do you personally know anyone who tested positive for COVID-19?”; (c) “Do you personally know anyone who died from COVID-19?”; and (d) “Has anyone from your family or close friends tested positive for COVID-19?” The sum of these four items created a composite score of exposure to COVID.

***Exposure to media coverage of COVID-19*** was tested by a single question: “Do you actively search for information about the development of the COVID-19 pandemic (e.g., read news websites, read updated news sites, listen to radio/TV, etc.) to learn about new developments?” Possible responses were “1. never or almost never”, “2. a few times a day”, or “3. all the time”.

***Media coverage-related stress*** was based on one item: “To what extent is the information you hear in the news calming vs. stressful?” Responses were rated on a 10-point scale, ranging from “1. very calming” to “10. very stressful.”

***Compliance with COVID-19 mitigation guidelines*** included six items with the following introduction: “To what extent do you adhere to the guidelines of the Ministry of Health in the following?” (e.g., quarantine, avoid attending events with many participants). Items and their distribution are presented in Table 1. Inter-item reliability (α Cronbach) was 0.71. A composite score averaging each participant’s scores on the six items was created. 

***Difficulties with adherence to COVID-19 mitigation guidelines*** included seven items, preceded by the statement: “These days, to what extent do you experience difficulties in the following situations?” (e.g., limiting the ability to walk around/go shopping/make arrangements on Passover eve). Items and their distribution are presented in Table 1. Inter-item reliability (α Cronbach) was 0.78. A composite score averaging each participant’s scores on the seven items was created.

***Economic constraints due to COVID-19*** were assessed by a single question: “To what extent has your financial situation been negatively affected by COVID-19?” Responses ranged from “1. not at all” to “5. to a great extent.”

### 3.6. Dependent Variables

***COVID-19-related concerns*** were assessed by seven items with the following introduction: “To what extent do you experience difficulties about each of the following statements regarding COVID-19?” (e.g., “Your family health status”). Responses were: “1. no difficulty”, “2. slight difficulty”, “3. quite a lot of difficulty”, or “4. a lot of difficulty”. Table 1 presents the items and their distributions. Inter-item reliability (α Cronbach) was 0.78. A composite score averaging each participant’s scores on the seven items was created.

***The need for help*** was calculated by the sum of two questions: First, “Following the outbreak of COVID-19 there are people who feel that they need help to overcome their experiences. Do you feel that *you* need help?” Responses were: “1. no”, “2. perhaps a little”, or “3. Yes, I do need help”. The second question was: “Have you considered seeking counseling due to personal, couple or family relationships difficulties?” Responses were: “1. not at all”, “2. perhaps a little”, “3. Yes, I do consider seeking counseling”, or “4. I am already in counseling”. Categories 3 and 4 were combined as “yes”. Inter-item reliability (α Cronbach) was 0.80. 

***Microaggressions*** were measured based on Nadal’s [41] microaggression scale. We constructed four items to reflect the expression of microaggression toward the ultra-Orthodox population during COVID-19 (e.g., “Someone else was making it clear that he is afraid of getting infected by me because I am an ultra-Orthodox Jew”). Participants were requested to indicate the number of times microaggression occurred to them during the COVID-19 pandemic on a scale ranging from “1. never” to “7. more than five times”. The items and their distributions are presented in Table 1. Inter-item reliability (α Cronbach) was 0.85. A composite score averaging each participant’s scores on the four items was created.

Age and gender were included as control variables in the multivariate analyses. Educational level and number of children in the family were not associated with any of the study variables and therefore were excluded from the analyses.

## 4. Data Analysis

Descriptive statistics were followed by bivariate Pearson correlations with all study variables. We then performed three hierarchical regression analyses, regressing the dependent variables (concerns, need for help, and reported microaggression) on the set of study variables and entered them into the regressions using four models in which any advanced model includes variables that were entered in the former models: 1. demographics; 2. exposure to COVID-19 and media coverage, and adherence to COVID-19 mitigation guidelines; 3. difficulties with adherence to COVID-19 mitigation guidelines, and stress related to media coverage; 4. financial constraints due to the COVID-19 pandemic. Regression analyses were conducted by the R program and treated the missing values with the method of full information maximum likelihood (FIML); [42].

## 5. Results

*Exposure to COVID-19*. Approximately 10% (10.3%) of the respondents had been in quarantine due to suspicion of COVID (n = 24, 9.5%) or a positive test (n = 2, 0.8%). About 80% knew at least one person (14.4% knew one person, and an additional 65.1% knew more than one person) who had tested positive for COVID. Thirty-two percent (32.4%) of the participants knew at least one person who had died from COVID-19 (17.2% knew one person, and 15.2% knew more than one person). More than forty percent (41.6%) reported that at least one family member or friend had tested positive (15.2% reported one person, and 26.4% reported more than one person). 

*Adherence to COVID-19 Mitigation Guidelines*. Table 1 shows that participants adhered less to the social distancing guideline (only 60% reported adhering “very much” to this guideline) and to quarantine (only 70.5% of the participants reported adhering “very much” to this guideline) than to other mitigation guidelines (e.g., 96.5% reported praying in a public *minyan* according to mitigation guidelines). We found it notable that the two guidelines least observed also had larger variance than other mitigation guidelines.

*Difficulties with compliance to the mitigation guidelines*. As seen in Table 1, the most common difficulties with COVID-19 mitigation guidelines were the closure of the schools/educational institutes (45.3% indicated “a lot of difficulty” in this regard) and celebrations with a limited number of guests (36.6% indicated “a lot of difficulty” in this regard).

*COVID-19-related concerns*. Both “lack of daily routine” and “community and family distancing” were reported as major concerns; 47% and 51.5%, respectively, reported “quite a lot” or “a lot of difficulty” with these aspects.

*Need for help*. One-fourth (25.5%) reported needing at least a little help with handling the COVID-19 crisis (22.0% reported “perhaps a little”, and 3.5% reported “yes, I need help”). More than one-fourth (27.0%) of the participants reported at least considering seeking counseling due to COVID-19 hardships (19.1% reported “perhaps a little”, 3.5% reported “yes, I do consider seeking counseling”, and 4.3% reported already being in counseling).

*Microaggression*. Although 36.0% of the respondents reported no experience of any act of COVID-related microaggression, one-third (33.1%) reported experiencing at least once that “someone assumed I had violated the rules of social isolation because I am an ultra-Orthodox Jew” and 35.1% reported experiencing at least once that “someone else was making it clear that he is afraid of getting infected by me because I am an ultra-Orthodox Jew”. The results are presented in Table 1.

*Associations between study variables*. Table 2 presents Means (and SD) of study variables and a matrix of the bivariate correlations. It shows that the dependent variables were correlated with some of the independent ones. COVID-related concerns were positively associated with difficulties with adherence to COVID-19 mitigation guidelines (*r* = 0.53, *p* < 0.01), stress related to media coverage (*r* = 0.37, *p* < 0.01), exposure to media coverage (*r* = 0.23, *p* < 0.01), and financial constraints from the pandemic (*r*= 0.29, *p* < 0.01). The need for help was positively associated with financial constraints from the pandemic (*r* = 0.34, *p* < 0.01) and had a negligible positive association with level of exposure to COVID-19 (*r* = 0.15, *p* < 0.05). Reported microaggression was positively associated with stress related to media coverage (*r* = 0.34, *p* < 0.01), difficulties with adherence to COVID-19 mitigation guidelines (*r* = 0.30, *p*< 0.01), and financial constraints from the pandemic (*r* = 0.24, *p* < 0.01).

*Prediction of COVID-related concerns, the need for help, and reported microaggression*. Table 3 presents the two advanced models (models 3 and 4) of the three hierarchical regression analyses for the dependent (predicted) variables: COVID-19-related concerns, need for help, and microaggression. For each dependent variable, the first model included demographic variables, and the second model added exposure to COVID-19 and media and adherence to COVID-19 guidelines (Models 1 and 2 are not shown in the table). Age and gender were not related to any of the dependent variables. In the second model, which added level of exposure to COVID-19, media coverage of the pandemic, and level of adherence to COVID-19 mitigation guidelines, we found that a higher level of exposure to COVID-19 was related to a greater need for help (*β* = 0.14, *p* < 0.05). Greater exposure to media coverage was associated with a higher level of COVID-related concerns (*β* = 0.25, *p* < 0.01). Adherence to COVID-19 mitigation guidelines was not significantly associated with any of the dependent variables. Overall, exposure variables explained 7.3%, 4.0%, and 2.9% of the variance for concerns, need for help, and microaggression, respectively. The third model added difficulties with adherence to COVID-19 mitigation guidelines and stress related to the media coverage; we found that greater difficulties with COVID-19 mitigation guidelines were positively associated with COVID-related concerns (*β* = 0.46, *p* < 0.01) and microaggression (*β* = 0.21, *p* < 0.01). Stress related to media coverage was also associated with COVID-related concerns (*β* = 0.29, *p* < 0.01) and microaggression (*β* = 0.32, *p* < 0.01). Overall, this model added to the explained variance 29.0% and 17.4% for concerns and microaggression, respectively, and did not add a significant percentage to the explained variance of need for help. The fourth and final model added financial constraints from the COVID-19 pandemic. We found that the financial constraints were positively associated with all three dependent variables. Greater financial constraints were associated with a higher level of COVID-related concerns (*β* = 0.14, *p* < 0.01), greater need for help (*β* = 0.34, *p* < 0.01), and a higher level of reported microaggression (*β* = 0.21, *p* < 0.05). The complete model explained 44.3%, 15.8%, and 23.9% of the variance of concerns, need for help, and microaggression, respectively.

## 6. Discussion

This study examined the rates of various aspects of COVID-19 experiences, such as level of exposure to COVID and difficulties in adherence to governmental mitigation guidelines, and whether those aspects predict COVID-related concerns, need for help, and reported microagression among ultra-Orthodox Jews in Israel.

*Level of Exposure*. The rate of personal exposure to COVID-19 was similar to the rate of other populations in Israel (Authors, in press). Nonetheless, the rates of friends or family members who had tested positive for COVID-19 (41.6%) were much higher among ultra-Orthodox Jews than among other populations in Israel (Authors, 2021). These differences may reflect the higher infection rate in that population compared to the general population in Israel [12], which was related to their high-density households [43]. The greater familiarity with people who tested positive for COVID-19 (80%) may reflect the ultra-Orthodox Jews’ collectivist identity and high cohesiveness [12].

*Difficulties in maintaining the mitigation guidelines and COVID-19-related concerns*. One of the most frequent difficulties mentioned was the limited number of guests allowed during celebrations. One of the strongest COVID-related concerns was “community and family distancing”. These difficulties and concerns may also reflect the collectivist and togetherness characteristics of ultra-Orthodox society. Religious activities take place in large communities and create a tight social network that facilitates face-to-face interactions, support, and a sense of belonging [13]. Such community religious activities and rituals help members to cope with numerous collective traumatic events, such as war and other acts of political violence [44,45]. In contrast, during the pandemic, the very same mechanisms that are usually considered to be helpful were considered harmful as they led to the spread of the virus [13]. This circumstance might have created a stress in itself, on top of the stress imposed by exposure to the pandemic, and might have fostered resistance to adherence to the guidelines, at least among part of the ultra-Orthodox population. It is worth mentioning that despite these difficulties, respondents reported a high rate of adherence to mitigation guidelines. The adherence rates are probably similar to those of the general Israeli population, although due to different measurement scales and types of analyses, numbers are hard to compare [46,47,48]. Thus, this study refutes Israeli public health opinion’s common perception that most ultra-Orthodox Jews did not comply with the governmental mitigation guidelines. Instead, these common perceptions may reflect another example of microaggression. 

*Perceived Microaggression*. Our findings suggest that a disaster may raise incidences of microaggression, at least according to the ultra-Orthodox perception. Specifically, in the face of COVID-19, two-thirds of this sample of ultra-Orthodox Jewish respondents reported at least one event of microaggression. These findings are supported by the existing literature indicating that disasters often escalate tensions and conflicts between groups, increasing racism and discrimination [49,50]. The COVID-19 pandemic intensified existing social conflicts between the ultra-Orthodox community and the majority of Israeli society, as the latter accused the ultra-Orthodox community of failure to adhere to the health mitigation guidelines and causing the virus to spread [18,51]. Such escalating tension may have long-lasting negative psychological, societal, and economic consequences long after the pandemic is over [18].

*The prediction of COVID-19-related concerns, need for help, and microaggression. COVID-related concerns* were predicted by the difficulty of adherence to mitigation guidelines, exposure to media, stress related to the media coverage, and financial constraints due to COVID-19. Thus, compliance with the mitigation guidelines took a heavy toll on ultra-Orthodox Jewish society, potentially due to the large gap created between their tight community lifestyle pre-COVID and the social distancing imposed by the governmental regulations [12]. The associations between adherence difficulties and concerns may also reflect growing mistrust in the governmental guidelines, a phenomenon that tends to characterize minority groups in general and the ultra-Orthodox Jewish community in particular [18]. Increasing the level of adherence to mitigation guidelines would require community empowerment and cultural sensitivity. Authorities should pay attention to individual risks and communities’ needs and resources and then negotiate the gaps between the benefits to the individual on the one hand, and community benefits and needs on the other [52]. Israeli national and local authorities made efforts to respect cultural norms and values when creating and executing COVID-19 regulations by seeking collaboration with religious leadership [18]. Nonetheless, our findings imply that adherence to guidelines was challenging and took a heavy toll on ultra-Orthodox Jewish society in terms of greater concerns. Stronger cultural sensitivity coupled with empowerment approaches might have alleviated their psychological distress. As in general society (Authors, in press), exposure to the media and stress related to the media coverage were also associated with COVID-related concerns. This finding supports previous studies revealing that intensified exposure to media coverage of disasters is often associated with increased stress reactions, worries, and deleterious health and mental health [23].

*The perceived need for help* was predicted (in the final model) only by financial constraints. These findings differ from other findings obtained by the authors in other contexts (Authors, 2010, 2014), in which needing help was associated with exposure to the stress agent (e.g., acts of terrorism) and psychological distress. This study’s results may reflect the financial disadvantages characterizing minority groups in general [4] and ultra-Orthodox Jews in particular [5]. These stressors were further intensified during the COVID-19 pandemic. Thus, when asked about needing help in the context of COVID-19, the most salient need this society acknowledged was the financial constraints. These findings also emphasize that COVID-19 is not just a health crisis but also a financial one [53].

*Microaggression* was predicted by difficulties with adherence to COVID mitigation guidelines, the stress related to the media coverage, and financial constraints. The association between microaggression and difficulties with adherence to COVID-19 guidelines may reflect the respondents’ feelings that their unique difficulties in compliance with the guidelines were ignored or misinterpreted [51], and that the guidelines were not culturally sensitive [18]. The relationship between stress related to the media coverage and microaggression may reflect the inherent tension between the media and the ultra-Orthodox community and the extensive media coverage of that community’s noncompliant behavior [24]. The media emphasized statistics showing the disproportionate rate of COVID infection and hospitalization among ultra-Orthodox society [54] but was not sensitive to the unique characteristics of this society, such as high-density households and limited access to information, which might partially explain the high rate of infection [8,43]. The ultra-Orthodox Jewish population felt that the media did not understand that the reason they were not complying with governmental guidelines was their strict adherence to religious practices, which according to their belief system is considered the most effective way to defeat disasters like the COVID-19 pandemic [49,55]. Thus, the media was perceived by ultra-Orthodox Jews as a partner involved in creating microaggression, or at least as its catalyst. These interpretations should be further explored.

## 7. Limitations

This study provides important insights regarding the concerns and needs of the ultra-Orthodox Jewish population in Israel during the COVID-19 pandemic, but its limitations should be noted. First, the study is based on a convenience sample and therefore does not represent the entire ultra-Orthodox population in Israel. In particular, the online data collection methods excluded part of the ultra-Orthodox population that does not access Internet or smartphones. Finally, this study relied on a cross-sectional design during the first wave of the pandemic. Thus, it does not cover the community’s needs and concerns during the second, more intense wave of the pandemic. Additional studies are needed to identify the possible trajectories of these needs and concerns over time. Future studies should address these limitations.

### Practical Implications

Our findings indicate a high level of perceived microaggression. Its risk factors were difficulties with adherence to COVID mitigation guidelines, the stress related to the media coverage, and financial constraints. Thus, Israeli society and the media have a moral obligation to understand the specific difficulties of this community to adhere to COVID-19 regulation, and the media should emphasize these difficulties whenever reporting on the ultra-Orthodox Jews violation of COVID-19 guidelines. In addition, the Israeli government needs to invest more resources into providing financial assistance to the Ultra-Orthodox society during this rough time and engaging this population in the efforts to reduce the spread of the pandemic. As mentioned before, ultra-Orthodox society, like other minorities, tends to distrust governmental authorities [18], but also tends to react favorably to noncoercive changes and guidelines from their reliable sources [56]. Thus, the government and law enforcement authorities should employ a more culturally sensitive approach that empowers the ultra-Orthodox community and promotes trust in local and governmental authorities [57]. Such an approach could include diversifying channels of communication and information to tap the traditional ways in which this community receives information. This may include closer collaboration with community leaders, using less formal paths of communication, and collaborating more effectively with the rabbis. Furthermore, attention should be paid to help mediate the gaps between this community and the larger society. Informing the public about the difficulties and conflicts experienced by this population could help reduce some of the animosity toward this group and reduce microaggression, with its debilitating long-term psychological and societal consequences.

## Figures and Tables

**Table 1:** Distribution of compliance with COVID-19 mitigation guidelines, difficulties in compliance with the guidelines, COVID-related concerns, and microaggression among Ultra-Orthodox Israelis (N = 225).

Adherence to COVID-19 Mitigation Guidelines								
	Not At All 1		Slightly 2		Moderately 3		Pretty Much 4	Very Much 5
	n %		n %		n %		n %	n %
Quarantine	4 2.3		2 1.2		9 5.3		36 21.1	119 70.5
Avoid contact such as shaking hands	2 0.8		2 0.8		4 1.6		13 5.3	222 91.4
Avoid attending large events	1 0.4		1 0.4		6 2.5		25 10.6	203 86.0
Maintain social distancing (at least 2m; 6 ft)	4 1.6		3 1.2		15 6.1		76 31.0	147 60.0
Pray in public (*minyan*) according to the mitigation guidelines	0 0.0		0 0.0		1 0.6		5 2.9	168 96.5
Comply with lockdown (when it occurred)	2 0.8		1 0.4		9 3.8		27 11.4	198 83.6
**Difficulties in Compliance with COVID-19 Guidelines**								
	**No Difficulty** **1**		**Slight Difficulty** **2**		**Quite a Lot of Difficulty** **3**		**A Lot of Difficulty** **4**	
	n %		n %		n %		n %	
Passover celebrations among close family only	96 39.8		73 30.3		45 18.7		27 11.2	
Your own/your husband’s/fa- ther’s/son’s inability to pray in a *minyan*	58 25.7		75 33.2		39 17.3		54 23.9	
Celebrations with limited number of guests	35 19.1		36 19.7		45 24.6		67 36.6	
Limitations on going out/shop ping/making arrangements for holidays	23 9.5		67 27.8		78 32.4		73 30.3	
Closure ofschools/educational Institutes	25 10.7		52 22.2		51 21.8		106 45.3	
Inability to leave the house on a holiday	25 10.2		73 29.9		65 26.6		81 33.2	
Neighborhood lockdown	37 23.4		47 29.7		30 19.0		44 27.9	
**COVID-Related Concerns**								
	**No Difficulty** **1**		**Slight Difficulty** **2**		**Quite a Lot of Difficulty** **3**		**A Lot of Difficulty** **4**	
	n %		n %		n %		n %	
Your health status	115 46.1		93 37.7		26 10.5		14 5.7	
Your family health status	49 19.8		101 40.9		63 25.5		84 13.8	
Loneliness	180 53.3		49 20.1		40 16.4		25 10.2	
Lack of daily routine	40 16.1		92 36.9		77 30.9		40 16.1	
Community and family dis- tancing	23 9.4		96 39.2		81 33.1		45 18.4	
My financial status	77 31.4		91 37.4		44 18.0		33 13.5	
**Microaggression**								
	**Never** **1**	**Once** **2**	**Twice** **3**	**Three Times** **4**	**Four Times** **5**	**Five Times** **6**	**More Than Five Times** **7**	
	n %	n %	n %	n %	n %	n %	n %	
Someone else made it clear that he was afraid of getting infected by me because I am ultra-Orthodox	157 64.9	16 6.6	20 8.3	12 5.0	10 4.1	3 1.2	24 9.9	
Someone assumed I had violated the rules of social isolation because I am ultra-Orthodox	160 66.9	21 8.8	16 6.7	14 5.9	9 3.8	3 1.3	16 6.7	
Someone told me that all ultra-Orthodox do not comply with the Ministry of Health’s mitigation guidelines	108 45.6	43 18.1	17 7.2	16 6.8	10 4.2	5 2.1	38 16.0	
Someone told me that because of the ultra- Orthodox, we will all be infected by COVID-19	143 59.3	32 13.3	16 6.6	15 6.2	3 1.2	4 1.7	28 11.6	

* *p* < 0.05, ** p < 0.01, *** p < 0.001.

**Table 2:** Means, standard deviations, and correlations of the study variables.

Variable	*M*	*SD*	1	2	3	4	5	6	7	8
1 COVID-related concerns	2.20	0.61								
2 Need for help	2.67	1.08	0.38 **							
3 Microaggression	2.37	1.70	0.18 **	0.17 **						
4 Exposure to COVID	6.75	1.92	0.06	0.15 *	0.03					
5 Exposure to media coverage	2.17	0.70	0.23 **	0.10	0.02	0.13				
6 Adherence to COVID-19 mitigation guidelines	4.70	0.50	−0.09	−0.08	−0.01	0.01	0.15 *			
7 Difficulties with adherence to COVID-19 mitigation guidelines	2.63	0.67	0.53 **	0.05	0.30 **	0.02	0.08	−0.16 *		
8 Stress related to the media coverage	5.71	1.92	0.37 **	0.09	0.34 **	−0.05	0.24 **	0.03	0.29 **	
9 Economic constraints due to COVID-19	0.76	0.86	0.29 **	0.34 **	0.24 **	0.07	0.04	0.06	0.06	0.06

* *p* < 0.05, ** p < 0.01, *** p < 0.001.

**Table 3:** Hierarchical regression analyses for predictors of psychological distress: COVID-related concerns, need for help, and microaggression among ultra-Orthodox Israelis.

	COVID-Related Concerns			Need for Help			Microaggression		
Predictor	*b*	*b* 95% CI [LL, UL]	*beta*	*b*	*b* 95% CI [LL, UL]	*beta*	*b*	*b* 95% CI [LL, UL]	*beta*
Model 3 : ᵃ Difficulties and stress									
Age	−0.00	[−0.01, 0.01]	−0.02	−0.00	[−0.02, 0.01]	−0.05	0.02	[0.00, 0.04]	0.13 *
Gender (female)	0.05	[−0.09, 0.20]	0.04	0.08	[−0.23, 0.40]	0.04	−0.50	[−0.94, 0.05]	−0.14 *
Exposure to COVID-19	0.01	[−0.02, 0.05]	0.04	0.08	[0.01, 0.16]	0.15 *	0.03	[−0.08, 0.14]	0.03
Exposure to media coverage	0.14	[0.04, 0.24]	0.16 **	0.13	[−0.10, 0.35]	0.08	−0.26	[−0.58, 0.06]	−0.10
Adherence to COVID-19 mitigation guidelines	−0.06	[−0.19, 0.08]	−0.05	−0.18	[−0.47, 0.12]	−0.08	−0.01	[−0.43, 0.41]	−0.00
Difficulties with adherence to COVID-19 mitigation guidelines	0.42	[0.31, 0.52]	0.46 **	0.01	[−0.21, 0.24]	0.01	0.52	[0.19, 0.85]	0.21 **
Stress related to the media coverage	0.06	[0.03, 0.10]	0.20 **	0.04	[−0.04, 0.12]	0.07	0.29	[0.17, 0.41]	0.32 **
	*R*^2^ = 0.361 **			*R*^2^ = 0.045			*R*^2^ = 0.203 **		
	Δ*R*^2^ = 0.288 **			Δ*R*^2^ = 0.005			Δ*R*^2^ = 0.174 **		
Model 4: Economic constraints									
Age	−0.00	[−0.01, 0.00]	−0.05	−0.01	[−0.02, 0.00]	−0.09	0.02	[−0.00, 0.04]	0.10
Gender (female)	0.05	[−0.08, 0.19]	0.04	0.12	[−0.18, 0.42]	0.05	−0.46	[−0.90, 0.01]	−0.13 *
Exposure to COVID-19	0.01	[−0.02, 0.04]	0.03	0.07	[−0.00, 0.14]	0.12	0.02	[−0.09, 0.13]	0.02
Exposure to media coverage	0.13	[0.03, 0.22]	0.14 **	0.13	[−0.08, 0.34]	0.08	−0.25	[−0.57, 0.07]	−0.10
Adherence to COVID-19 mitigation guidelines	−0.09	[−0.22, 0.04]	−0.07	−0.21	[−0.49, 0.07]	−0.10	−0.03	[−0.45, 0.39]	−0.01
Difficulties with adherence to COVID-19 mitigation guidelines	0.40	[0.30, 0.50]	0.44 **	−0.01	[−0.23, 0.20]	−0.01	0.50	[0.18, 0.82]	0.20 **
Stress related to the media coverage	0.06	[0.02, 0.09]	0.18 **	0.03	[−0.05, 0.11]	0.05	0.28	[0.17, 0.40]	0.31 **
Economic constraints due to COVID-19	0.10	[0.00, 0.19]	0.14 *	0.42	[0.21, 0.63]	0.34 **	0.41	[0.10, 0.72]	0.21 **
	*R*^2^ = 0.443 **			*R*^2^ = 0.158 **			*R*^2^ = 0.239 **		
	Δ*R*^2^ = 0.082 **			Δ*R*^2^ = 0.113 **			Δ*R*^2^ = 0.036 **		

* *p* < 0.05. ** *p* < 0.01. ᵃ *R*² for Models 1 and 2, for the three dependent variables, respectively, are: Model 1, *R*² = 0.000, 0.003, 0.027. Model 2, *R*² = 0.073 **, 0.040, 0.029.

## Data Availability

The data presented in this study are available on request from the corresponding author. The data are not publicly available due to privacy issues.
